# Program Development and Effectiveness of Workplace Health Promotion Program for Preventing Metabolic Syndrome among Office Workers

**DOI:** 10.3390/ijerph14080878

**Published:** 2017-08-04

**Authors:** Hosihn Ryu, Jiyeon Jung, Jeonghyun Cho, Dal Lae Chin

**Affiliations:** 1 College of Nursing, Korea University, Seoul 02841, Korea; hosihn@korea.ac.kr; 2 Department of Nursing, Inje University, Busan 47392, Korea; jhcho@inje.ac.kr; 3 School of Nursing, University of California, San Francisco, CA 94143, USA; dal.chin@ucsf.edu

**Keywords:** metabolic syndrome, office workers, intervention program, effectiveness

## Abstract

This paper aims to develop and analyze the effects of a socio-ecological model-based intervention program for preventing metabolic syndrome (MetS) among office workers. The intervention program was developed using regular health examinations, a “health behavior and need” assessment survey among workers, and a focus group study. According to the type of intervention, subjects took part in three groups: health education via an intranet-based web magazine (Group 1), self-monitoring with the U-health system (Group 2), and the target population who received intensive intervention (Group 3). The intervention programs of Group 1 and Group 2, which relied on voluntary participation, did not show significant effects. In Group 3, which relied on targeted and proactive programs, showed a decrease in waist circumference and in fasting glucose (*p* < 0.001). The MetS score in both males (−0.61 ± 3.35 versus −2.32 ± 2.55, *p* = 0.001) and females (−3.99 ± 2.05 versus −5.50 ± 2.19, *p* = 0.028) also showed a statistically significant decrease. In light of the effectiveness of the intensive intervention strategy for metabolic syndrome prevention among workers used in this study, companies should establish targeted and proactive health care programs rather than providing a healthcare system that is dependent on an individual’s voluntary participation.

## 1. Introduction

Metabolic Syndrome (MetS) is a cluster of several metabolic abnormalities that are risk factors associated with coronary artery disease. Thus, MetS has been shown to increase death rates associated with diabetes mellitus (DM) and cardiovascular diseases (CVDs) [1,2,3,4]. The prevalence of MetS in Korea is increasing along with an increasing rate in the worldwide population [5,6,7]. Of particular concern, due to DM and CVDs, is the death rate of the adult population in Korea, which is reported to be higher than the Organization for Economic Co-operation and Development (OECD) average [8].

About 65.9% of adults in Korea are employed [9] and Korea ranks third for the longest hours worked among OECD countries [10]. In addition to bearing heavy workloads, Korean office workers experience stress, smoke, drink frequently due to work dinners, and have less physical activity than other types of workers due to the sedentary nature of their work [11,12]. Due to these work-related risk factors, high MetS rates have been reported among Korean office workers [13,14]. This means that most of the adult population in Korea is exposed to working conditions that place them at risk of chronic disease. Even though improving health behavior for the prevention and management of MetS is important [15,16], a structured Workplace Health Promotion Program (WHPP) is offered in very few companies in Korea. 

The comprehensive WHPP has been proposed by The American Heart Association (AHA) for the prevention of cardiovascular disease [17]. The Centers for Disease Control and Prevention has asserted that the use of effective workplace programs and policies can reduce health risks such as hypertension (HTN), dyslipidemia, and DM, which improves the quality of life for workers [18]. The CDC has promoted the WHPP as a planned, organized, and comprehensive set of programs, policies, benefits, and environmental supports based on workers’ needs. Thus, workplaces may provide an important and unique opportunity for promoting the adult population’s health [19,20].

For an effective WHPP, a socio-ecological approach that considers the many factors impacting individuals’ health behavior is necessary [21]. The intervention program was developed using the theoretical framework derived from the Socio-ecological Model (SEM) to prevent MetS among Korean office workers. The SEM is based on the premise that an individual’s health behavior is affected by various external systems and organizations; policies should take into account not only personal characteristics but also interpersonal, organizational, social, and environmental factors [22]. In the theoretical view provided by the SEM, it is crucial to identify not only the influence of individual factors on workers’ health but also that of the work environment. In order to see substantial effects from WHPP, it is necessary to provide a comprehensive program that includes workers’ participation, peer-to-peer support, and the development of a healthy work environment. Based on the SEM, this intervention program selected variables containing organizational and environmental factors, in addition to personal demographic, psychological, and lifestyle variables.

In this study, a MetS prevention–intervention program among office workers was developed utilizing the SEM. The results from the program will be used as a basis to establish a workplace health management system. To this aim, this study introduces the development and implementation of intervention strategies and its verified effects.

## 2. Materials and Methods

### 2.1. Study Design and Setting

In this study, we present the development and effects of a SEM-based intervention program for preventing MetS among office workers. Due to the study’s quasi-experimental setup, a pre-test and post-test design was used. This intervention study was conducted among 891 office workers employed at a single firm in Seoul, South Korea.

### 2.2. Program Development 

Four steps were taken in order to develop an effective intervention program. First, we conducted a 10-year cohort analysis of regular health examinations to assess the MetS indicators of office workers. In the case of male office workers, the results showed that the risk factors fasting glucose (FG), triglycerides (TG) and waist circumference (WC) significantly increased with working years [23]. Second, we conducted a factor analysis of the SEM variables to identify risk factors of MetS among the workers [24].

Third, we conducted a health behavior and need assessment survey issued via the firm’s intranet to determine appropriate intervention strategies. In order to implement a prevention program that meets the needs of workers at the firm, we examined topics such as WHPP awareness, one’s participation experience, why they participated, and what information they sought (response rate 36.1%). We found that more than 50% of respondents answered “I don’t know” about WHPP, and about 30% reported no experience. The most common reason for participating was “weight management”. There was relatively high interest in monitoring one’s health through the U-health system. In this study, the U-health system allows individuals to measure their own health information at the firm’s on-site health clinic and a specially designed electronic device (“CADY U-Health Zone PRO STANDARD”) allows individuals to access their information through a kiosk or personal computer using a personal ID. Thus, this system allows for continuous self-monitoring of one’s own health.

Fourth, a focus group study (FGS) was conducted, composed of personnel managers, the company-affiliated occupational health nurse, and researchers. The FGS helped to improve managers’ awareness and create an intra-organizational support system, providing a foundation to create an intervention program that could be implemented on site. Following the FGS, detailed information about the intervention program was provided at the company’s labor council meeting and an agreement was reached to commit to the intervention program. 

**Figure 1:**
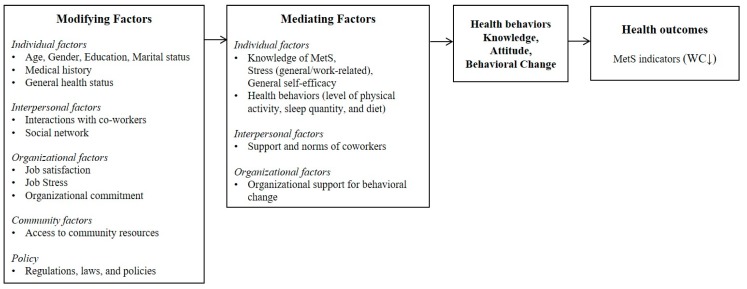
Intervention framework of the study.

The development and implementation process of this prevention program was based on the theoretical framework of the SEM, in which individuals’ health behavior is not only affected by individual characteristics but is also affected by various external factors [25]. Thus, it is important to consider interpersonal, organizational, environmental, and overarching policy factors. Figure 1 presents the variables used in this study reflect the SEM. 

### 2.3. Implementation Process

In this study, three prevention programs were implemented (Table 1). In the first program (Group 1, the “health education group”), targeting all office workers in the firm, health education was distributed via an intranet-based web-magazine. The second program provided further health education to voluntary participants (Group 2, the “self-monitoring group”), who could use the U-health system to self-monitor one’s health. These programs were provided to Group 1 and Group 2 for a total of 6 months (from November 2013 to April 2014). In the third program, subjects were the target population who had over three MetS indicators or a BMI (Body Mass Index, defined as the body mass divided by the square of the body height) greater than 25 kg/m^2^. For this “intensive intervention group” (Group 3), the program was provided for a total of 10 weeks from (November 2013 to January 2014).

For Group 1, information on health promoting behavior was disseminated by a web-magazine leaflet, provided once every two weeks. This health education included MetS-related diseases, as well as understanding the impact of chronic stress, obesity, nutrition, exercise, drinking, and smoking on MetS. Though the relative ease of distribution and production of material on health education via a Web-magazine has its advantages, the material was provided in a manner that depends on voluntary participation. We could only gauge use by total hits and thus could not accurately assess employees’ understanding or the application of its contents. 

Group 2 used the health clinic’s U-Health System managed by the occupational health nurse. Through the U-health system, one can voluntarily measure health indicators, and the nurse can provide consultation regarding the measurement results and any changes. A total of 180 people participated in this six-month-long prevention program. The advantages of the U-Health System are that individuals’ health information is guaranteed to be kept confidential and that all one’s health information can be self-monitored. This program has an inherent disadvantage because it too is dependent on individuals’ voluntary participation and interest; we cannot obligate participation.

An intensive strategy was used for Group 3 (target population) by comprehensive management with the organizational support. In this program, subjects were able to improve their athletic ability through the strengthening of basic physical fitness and form supportive bonds between team members through group exercise classes. The program was performed under the guidance of two certified exercise trainers for one hour, two times a week (for a total of 20 times). Additionally, structured exercises and diet plans were made available in order to achieve a balanced body composition and reduce central obesity (WC). Considering accessibility and usability, an on-site exercise program was conducted shortly after office hours. In addition, in order to promote daily physical activity and encourage lasting healthy behaviors, participants utilized a handheld daily activity tracker (“TANITA FB-730”). The data was collected and patterns of physical activity could be self-monitored by this individualized, wearable electronic device. The data on one’s physical activity is stored on the tracker itself and can be uploaded so that employees can check their progress easily. By continuously monitoring subjects’ participation rates in the program and their physical activity, the occupational health nurse could give individual fringed health counseling. The counseling was offered with flexible schedules if the participant requested.

This intervention program was approved by the Institutional Review Board (IRB, 1040548-KU-IRB-13-12-A-2). Permission was also obtained from the president of the company, the director of the occupational health center in the company, and participants of this study.

### 2.4. Measurement

For Group 1 and Group 2, we used MetS indicators (obtained by BP measurement, anthropometric measurement and blood tests) from regular health examinations. We analyzed the health examinations results from 2013 (pre-intervention) and 2014 (post-intervention), which were conducted at the company-designated general hospital, in accordance with the Korean Occupational Safety and Health Act. We also collected health screening questionnaire data which was checked just before the health examination. For Group 3, additional health examinations were given pre- and post-intervention. Also, in order to understand subjects’ individual and organization-related health behavioral characteristics, we conducted intranet-based health behavioral surveys between April and June 2013 (pre-intervention) and May and August 2014 (post-intervention). 

Measured MetS indicators from Group 1 and Group 2’s regular health examinations included those obtained from blood tests (FG, HDL-C, and TG), anthropometric measurements (WC), and a blood pressure (BP) measurement. MetS indicators were based on the criteria set by The National Cholesterol Education Program Adult Treatment Panel III (NCEP-ATP III) [26]. We based the WC on the lower criteria set by the Korean Society for the Study of Obesity (KSSO), which takes into account the body type of Korean people [27]. Of the five MetS indicators (WC, BP, FG, TG, HDL-C), if three or more of the following conditions are met, subjects were defined as having MetS: WC over 90 cm (male), 85 cm (female); BP over 130/85 mmHg or medicated; FG over 100 mg/dL or medicated; TG over 150 mg/dL or medicated; or HDL-C less than 40 mg/dL (male), 50 mg/dL (female) or medicated.

Through the Intranet-based health behavioral survey, we were able to examine the SEM variables which included individual factors and organizational factors of health behavioral characteristics. The individual factors examined were general health status, general self-efficacy, stress level, knowledge of MetS, and health behaviors including physical activity, sleep quantity, and diet. To measure general health status, subjects were asked to rate their general perception of their own health using a five-point Likert response scale. A 10-item scale was used to assess a general sense of perceived self-efficacy [28] and each item used a four-point Likert response scale. The reliability in its development was represented as Cronbach’s alpha coefficients for 0.75, and 0.89–0.90 in this study. For Stress level, respondents rated both their general stress level and work-related stress level on a scale from 1 (low) to 7 (high) points. To measure knowledge of MetS, we took the sum score from a total of six questions that assessed respondents’ MetS-related health knowledge where subjects were asked to ascertain whether statements made about MetS were true or false. The Cronbach’s alpha coefficients of knowledge of MetS was 0.70–0.79. Physical activity was measured by the number of times a week that a respondent participated in at least 30 minutes of vigorous physical activity. For sleep quantity, two response choices were given: 7–8 h (good) or something else (fair). To measure diet, we summed the scores of 10 questions that asked about subjects’ eating habits with response choices being yes (over five times a week) or no (less than four times a week). The Cronbach’s alpha coefficients of diet was 0.62–0.79. Organizational factors were examined using three variables: organizational commitment, job satisfaction, and job stress. Organizational commitment was assessed using a four-point Likert scale in response to two statements and was calculated as one variable by summing these ratings. A higher score indicated greater commitment to the organization [29]. In this study, the Cronbach’s alpha coefficients of organizational commitment were 0.60–0.74. Job satisfaction was measured using a single question with responses ranging from very dissatisfied to very satisfied. Job stress was measured using the Korean Occupational Stress Scale-Short Form (KOSS-SF) [30]. The KOSS-SF consists of 7 subscales with 24 items: job demands (4 items), insufficient job control (4 items), inadequate social support (3 items), job insecurity (2 items), organizational system (4 items), lack of rewards (3 items), and occupational climate (4 items). Each question used a four-point Likert response scale and the sum of the scores for each subscale was converted to a 100-point system. The reliability in its development was represented as Cronbach’s alpha coefficients of 0.51–0.82. In this study, the internal reliability coefficient was 0.66–0.67.

In the case of Group 3’s intensive intervention program, for participants who received extra health examinations pre- and post- intervention we measured MetS indicators including WC, and BP. We also measured FG, HDL-C, and TG which uses peripheral blood. The medical lab technicians performed the hematologic exam using a portable clinical chemistry analyzer (“SAMSUNG LABGEO^pt10^”). The measured MetS indicators were used to calculate a MetS score, or continuous metabolic syndrome score [31]. This MetS score is calculated by summing the values of the z-scores of five MetS indicators. A lower score indicates a positive effect of the intervention. The MetS was calculated as follows: In the case of men, z-score = [(40 − HDL-C)/9.0] + [(TG − 150)/81.0] + [(FG − 100)/11.3] + [(WC − 90)/7.7] + [(MAP * − 100)/9.1], and for women, z-score = [(50 − HDL-C)/14.1] + [(TG − 150)/81.0] + [(FG − 100)/11.3] + [(WC − 85)/9.0] + [(MAP − 100)/9.1]. Mean Arterial Pressure * (MAP) was calculated as “Diastolic BP+1/3 × (Systolic BP − Diastolic BP)”. 

### 2.5. Statistical Analysis

The collected data was analyzed using the IBM SPSS statistical package (ver. 21; sSPSS Inc., Chicago, IL, USA). Frequencies, percentages, means and standard deviations are presented for each of the general characteristics for each group of subjects. In the descriptive analysis of the indicators, the mean and standard deviations are shown for the continuous variables and the frequencies and percentages are shown for the categorical variables. To test the effect of the intervention program for each of the groups, we conducted *t*-tests for continual variables and McNemar tests for categorical variables. The level of statistical significance was defined as a *p*-value < 0.05.

## 3. Results

Of the company’s 891 employees, subjects included in the final analysis were those who received pre- and post-intervention general health examinations (for Groups 1 and 2) and extra health examinations (for Group 3) as well as respondents to the intranet-based health behavioral survey: Group 1 (*n* = 449, 50.4% indicate the response rate of those who completed both health examinations and the survey among the participants in each group), Group 2 (*n* = 75, 41.7%), Group 3 (*n* = 41, 66.1%). Results for the general characteristics and demographics for each group are found in Table 2.

### 3.1. Program Effectiveness for Group 1 and Group 2

Upon the completion of the intervention program, we observed no significant positive changes in MetS indicators for both Groups 1 and 2. Also, there was no significant change in the SEM variables in Group 1. In Group 2, there was a significant increase in the mean diet score in Table 3. 

### 3.2. Program Effectiveness for the Target Population (Group 3)

After the intervention, there was a significant reduction in the mean WC (89.96 ± 9.87 vs. 86.93 ± 9.79) and FG (93.44 ± 11.78 vs. 84.56 ± 9.55) in Group 3 (*p* < 0.001). Also, as a result of the intensive intervention program, the number of subjects with each of the MetS indicators was either reduced (WC, BP, FG, HDL-C) or stayed the same (TG). In particular, the mean decrease of WC, as well as the corresponding WC risk factor condition and elevated waist circumference, showed a statistically significant decrease (*p* = 0.002). MetS prevalence decreased by about 9.8% between pre-intervention (*n* = 17, 41.5%) and post-intervention (*n* = 13, 31.7%). The MetS score (MetS risk converted into a continuous z-score) showed a significantly decreased mean for both men (−0.61 ± 3.35 vs. −2.32 ± 2.55, *p* = 0.001) and women (−3.99 ± 2.05 vs. −5.50 ± 2.19, *p* = 0.028), which indicates that the intensive intervention program improved each MetS indicator and resulted in a reduction in the prevalence of MetS, in Table 4. 

In addition, among the SEM variables of health behavioral characteristics there was an improvement in individual factors such as general health status, general self-efficacy, stress level, knowledge of MetS, physical activity level, sleep quantity, and diet score between pre- and post-interventions. However, these changes were not statistically significant. Among the changes of the job stress component, such as job demand, insufficient job control, and lack of rewards, only job demand showed a statistically significant reduction (*p* = 0.047).

## 4. Discussion

In this study, we developed, implemented, and examined the effect of workplace-based MetS prevention programs. The theoretical framework of the interventions was developed using the SEM, which comprehensively takes into account individual, organizational and environmental variables that affect one’s health behavior. We attempted three different intervention strategies. The results did not show significant improvement in MetS indicators in Group 1 and Group 2, who both received a passive intervention dependent on voluntary participation. However, Group 3, who received the intensive intervention accomplished with the company’s systematic support and management, showed a statistically significant reduction between pre- and post-intervention WC and FG. Further, the MetS score’s post-intervention means for both men and women significantly decreased. In sum, a tailored and intensive WHPP, targeted at the at-risk population, based on active participation, and implemented with the organization’s systematic support and management, was effective. 

The positive results among the at-risk population with an intensive intervention were consistent with previous literature. For example, male workers diagnosed with MetS (*n* = 37) in Takimoto et al.’s study, which reported improvement in body weight (BW), BMI, FG, HbA1c, and insulin levels for the treatment group [32]. In Maruyama et al.’s study, MetS male office workers who had risk factors for MetS (*n* = 26) were given an exercise program, diet improvement education and counseling, which also resulted in an improvement in each of BW, BMI, FG, and insulin levels [33]. A workplace-based exercise program showed similar changes in BW, body fat percentage, WC, TG, total cholesterol (TC), HDL-C, and LDL-C for male workers who have risk factors for MetS (*n* = 26) in the study conducted by Matsuo et al. [34]. Further, through a worksite intervention program that included the provision of education, reform of eating habits, the promotion of physical activity, and counseling, overweight workers in Mache et al.’s study increased physical activity, improved their diet, and saw a decrease in BMI [35]. Moreover, though subjects may already be diagnosed with MetS, an intensive intervention tailored to those with risk factors was found to be generally effective in improving BW and BMI and was effective in improving MetS indicators including WC. These results have been consistently corroborated. 

On the other hand, WHPP, a passive-based intervention which is reliant on voluntary participation, had a relatively weak effect. In Engbers et al.’s systematic review of environmental modifications strategies in WHPP, research studies found no evidence of improvement for health risk factors, including cholesterol levels, BMI, and BP [36]. Further, Lemon et al. also suggested that when there is organized and systematic management support for the voluntary participation-based WHPP, one could expect a larger effect from the program [37]. That is, when an active, intensive intervention program is implemented and takes into account individual and organizational factors, it can be considered more effective for workers’ health promotion [17,38].

This study has several strengths. First, the results from the development and implementation of intervention programs using three different strategies for the prevention of MetS among office workers were presented. In this study, the WHPP’s success suggests the importance of an intensive intervention program implemented with the systematic support and management of the company. Second, in the process of developing and implementing these intervention programs based on the SEM, we actively sought to reflect subjects’ needs (individual factors) and to elicit the support of colleagues and the organization (organizational factors). Moreover, this study tried to reflect important considerations of WHPP: workers’ privacy and flexibility. For example, the individual health counseling was offered with flexible schedules when the participant requested and was based on their regularly monitored health status. This study’s development process and results can be used as a good example for the development and implementation of WHPP in Korea. Third, this study emphasized the occupational health nurse’s role as responsible for the workers’ health as well as the important health care provider. 

On the other hand, there are some limitations of this study. First, this study was conducted using a non-randomized, quasi-experimental design where subjects were recruited on a voluntary basis, which may have led to confounding bias. Furthermore, this research was only conducted within group comparisons that would not reflect the significance of 3-group design. The SEM composed of individual, organizational and environmental variables, which is the theoretical framework used in this study, affects workers’ health status. From this perspective, organizational and environmental strategies that affect the whole company members were the key components of the implementation programs. Because of the diffusion problems with effectiveness of these strategies, the study was not able to conduct implementation with three independent groups that can conduct analytic comparisons between groups. Second, there were some statistically significant changes in SEM variables in Group 3 (e.g., job demand). It is hard to explain that the change among job stress variables is not consistent. However, it is possible to suppose that the strategy contains motivated participation, peer group, and organizational support in intensive intervention, possibly leading to a decrease in job demand. Third, because this research was not carried out by the government but by private researchers through a 3-year research project, we experienced considerable difficulty and effort was required for change in the corporate organization and workers’ health behavior. Even after the completion of the intervention programs, workers recognized the need for an ongoing WHPP. Thus, in regard to the health promotion of workers, an important achievement of this study is changing perceptions so that the occupational health nurse can continue the WHPP. Finally, the study sample included only office workers in a single firm of a country and the sample sizes of each group were quite different, which may have limited the generalizability of the findings. In the future, we suggest repeated and continuous research based on large populations in various settings.

## 5. Conclusions

In conclusion, this study shows that the organization’s active support and systematic management of at-risk individual’s strongly motivated participation and is very important for the implementation of WHPP for the prevention of MetS among workers. This research suggests that intensive interventions that are geared for the target population may be more effective than passive interventions that are dependent on workers’ voluntary participation. We anticipate that the expansion of MetS prevention programs in the workplace, using this study’s results, will be an opportunity to bring about a change in the public perception of the importance of health care for workers, which is currently a health care blind spot. 

## Figures and Tables

**Table 1:** Intervention subjects and the methods of the study.

Subjects’ Characteristics	Intervention Methods and Contents
Group 1 (*n* = 891; Health Education Group) Whole office workers in the firm	Health education Health education using Intranet (1 page-leaflets by webzine) for once in 2 weeks for 6 months (total 12 times) Health education contents consist of knowledge of MetS, and health behaviors: chronic stress, obesity, nutrition, physical activity, drinking behavior and smoking, etc.
Group 2 (*n* = 180; U-health System Group) Voluntary participant using U-health system	Health education + Using U-health system Using U-health system with personal health related goals: Blood Pressure; Body Weight; Body Mass Index; Body Fat Percentage or Waist Circumference etc. Regularly checking health indicators using U-Health System and motivating by occupational nurse for 6 months (recommendation for check more than 1 times per 2 weeks) Self-monitoring health indicators by PC with keeping the personal privacy of workersIndividualized health counseling about health status and trend by occupational health nurse
Group 3 (*n* = 62; Target Population Group) Workers with over 3 MetS indicators (or/and) BMI ≥ 25	Health education + Using U-health system + Intensive intervention program Intensive intervention program contents consist of group exercise for WC control; daily activity monitoring; health consultation A round runs for 1 h × 2 times (/a week) × 10 weeks, total 2 rounds (1st round *n* = 30, 2nd round *n* = 32) Intensive intervention program just after leaving work in workplace settings Intensive physical exercise program: “CORE training and cycling exercise program” in workplace by certificated exercise trainer Using handheld daily activity tracker to improve daily activity rate Personal health education and consultation by occupational health nurse from the company

**Table 2:** Baseline general characteristics of the study population.

Characteristics	Group 1 (*n* = 449) ^a^	Group 2 (*n* = 75) ^a^	Group 3 (*n* = 41) ^a^
	Mean ± SD or *n* (%)		
Age (Years)	35.31 ± 7.74	32.92 ± 7.07	36.49 ± 8.25
Gender			
Male	331 (73.7)	55 (73.3)	31 (75.6)
Female	118 (26.3)	20 (26.7)	10 (24.4)
Education			
High school or lower	25 (5.6)	2 (2.7)	5 (12.2)
College or University	359 (80.0)	70 (93.4)	34 (82.9)
Graduate school	65 (14.5)	3 (4.0)	2 (4.9)
Marriage status			
Unmarried	207 (46.2)	45 (60.0)	19 (46.3)
Married	241 (53.8)	30 (40.0)	22 (53.7)
Working type			
Management	106 (23.6)	27 (36.0)	11 (26.8)
Sales	343 (76.4)	48 (64.0)	30 (73.2)
Years worked on the job	7.85 ± 7.63	6.07 ± 7.27	9.73 ± 9.51

^a^ Numbers for characteristics do not total the same number due to missing data.

**Table 3:** Pre- and post-results of participants by regular health examinations (Group 1, Group 2).

Characteristics	Group 1 (*n* = 449) ^a^				Group 2 (*n* = 75) ^a^			
	Pre	Post	*t*	*p* ^c^	Pre	Post	*t*	*p* ^c^
	Mean ± SD or *n* (%) ^b^				Mean ± SD or *n* (%) ^b^			
Waist circumference (cm)	81.07 ± 10.72	81.41 ± 10.76	−1.479	0.140	82.09 ± 10.60	82.53 ± 10.02	−0.733	0.466
Blood pressure (mmHg)								
Systolic blood pressure	115.75 ± 12.80	116.40 ± 13.77	−1.229	0.220	116.10 ± 10.99	116.07 ± 12.84	0.022	0.982
Diastolic blood pressure	72.21 ± 9.62	75.13 ± 10.42	−6.433	<0.001	71.55 ± 7.89	73.73 ± 10.57	−2.080	0.041
Fasting glucose (g/dL)	93.28 ± 13.51	94.63 ± 18.00	−2.628	0.009	92.00 ± 9.39	94.85 ± 18.41	−1.344	0.183
Triglycerides (g/dL)	114.15 ± 92.63	119.63 ± 109.51	−1.546	0.123	100.00 ± 65.58	99.27 ± 68.98	0.127	0.900
HDL-cholesterol (g/dL)	55.60 ± 12.79	55.54 ± 13.47	0.160	0.873	55.88 ± 11.94	56.20 ± 12.51	−0.340	0.735
**MetS indicators prevalence**								
Elevated waist circumference	86 (20.5)	84 (20.0)		0.878	18 (25.7)	17 (24.3)		1.000
Elevated fasting glucose	85 (19.4)	97 (22.1)		0.195	9 (12.2)	14 (18.9)		0.302
Elevated triglycerides	91 (20.9)	107 (24.6)		0.068	9 (12.2)	11 (14.9)		0.727
Reduced HDL-cholesterol	62 (14.3)	58 (13.3)		0.672	9 (12.2)	8 (10.8)		1.000
Elevated blood pressures	94 (21.3)	124 (28.1)		0.002	15 (20.8)	17 (23.6)		0.804
**MetS prevalence**				0.551				0.508
Less than 3 components	360 (85.7)	355 (84.5)			62 (91.2)	59 (86.8)		
3 or more components *	60 (14.3)	65 (15.5)			6 (8.8)	9 (13.2)		
**Individual factors**								
General health status ^d^				0.207				0.791
Bad	207 (68.3)	219 (72.3)			38 (61.3)	40 (64.5)		
Good	96 (31.7)	84 (27.7)			24 (38.7)	22 (35.5)		
General self-efficacy	29.09 ± 3.72	29.65 ± 3.46	−2.796	0.006	30.00 ± 4.01	29.56 ± 3.39	0.945	0.348
Stress level (1–14)	9.02 ± 2.32	8.91 ± 2.17	0.952	0.342	8.44 ± 2.15	8.45 ± 1.86	−0.075	0.940
General (1–7)	3.96 ± 1.37	3.86 ± 1.36	1.259	0.209	3.53 ± 1.28	3.45 ± 1.17	0.567	0.573
Work-related (1–7)	5.06 ± 1.26	5.04 ± 1.26	0.183	0.855	4.90 ± 1.33	5.00 ± 1.17	−0.704	0.484
Knowledge of MetS (0–6)	1.73 ± 1.77	1.51 ± 1.54	2.365	0.018	1.81 ± 1.59	1.52 ± 1.52	1.302	0.197
Physical activity (per week)				0.315				0.542
No	186 (41.4)	187 (41.6)			29 (38.7)	26 (34.7)		
1–2 times	171 (38.1)	186 (41.4)			29 (38.7)	31 (41.3)		
≥3 times	92 (20.5)	76 (16.9)			17 (22.7)	18 (24.0)		
Sleep quantity (h) (3–10)				0.141				-
Others	213 (70.5)	225 (74.5)			39 (62.9)	40 (64.5)		
Good sleep (7 or 8 h)	89 (29.5)	77 (25.5)			23 (37.1)	22 (35.5)		
Diet (scores) (0–10)	4.89 ± 2.30	5.12 ± 3.02	−1.481	0.140	4.69 ± 2.54	5.81 ± 3.97	−2.358	0.022
**Organizational factors**								
Organizational commitment	6.08 ± 1.06	6.07 ± 0.95	0.235	0.815	6.15 ± 0.97	5.82 ± 0.80	2.224	0.030
Job satisfaction ^e^				0.470				0.503
Not satisfied	145 (48.5)	154 (51.5)			31 (50.0)	35 (56.5)		
Satisfied	153 (51.2)	146 (48.8)			31 (50.0)	27 (43.5)		
Job stress								
Job demand	54.27 ± 12.38	54.18 ± 11.41	0.107	0.915	55.26 ± 11.64	56.87 ± 11.90	−0.894	0.375
Insufficient job control	56.65 ± 14.90	57.95 ± 14.57	−1.514	0.131	57.31 ± 15.36	57.16 ± 15.06	0.079	0.938
Inadequate social support	64.39 ± 15.60	64.35 ± 14.23	0.038	0.970	60.43 ± 18.43	58.48 ± 16.34	0.980	0.331
Job insecurity	42.52 ± 22.55	47.94 ± 23.04	−3.367	0.001	41.52 ± 20.20	51.46 ± 24.25	−2.572	0.013
Organizational system	54.36 ± 15.98	54.77 ± 14.20	−0.464	0.643	52.49 ± 16.66	54.53 ± 16.82	−0.952	0.345
Lack of rewards	57.40 ± 16.25	56.69 ± 14.50	0.773	0.440	55.36 ± 18.24	57.50 ± 16.68	−0.900	0.372
Occupational climate	36.13 ± 17.13	37.84 ± 17.73	−1.564	0.119	39.91 ± 19.21	42.54 ± 20.64	−0.828	0.411

SD = Standard deviation; BMI = Body mass index; HDL-cholesterol = high-density lipoprotein cholesterol; MetS = metabolic syndrome; * Metabolic syndrome was defined as having at least three of five components; ^a^ Numbers for characteristics do not total the same number due to missing data; ^b^ Paired *t*-tests for continual variables between groups; McNemar tests were conducted for categorical variables between groups; ^c^ *p* value for McNemar test or paired *t*-test; ^d^ Bad: very poor, poor, fair; Good: good, very good; ^e^ Not satisfied: Very dissatisfied, dissatisfied, fair; Satisfied: satisfied, very satisfied.

**Table 4:** Pre- and post-results of the target population group (Group 3, *n* = 41) ^a^.

Characteristics	Pre-Intervention	Post-Intervention	*t*	*p* ^c^
	Mean ± SD or *n* (%) ^b^			
Waist circumference (cm)	89.96 ± 9.87	86.93 ± 9.79	−4.363	<0.001
Blood pressure (mmHg)				
Systolic blood pressure	126.13 ± 12.68	125.05 ± 13.49	−0.596	0.555
Diastolic blood pressure	84.68 ± 10.91	85.13 ± 10.75	0.329	0.744
Fasting glucose (g/dL)	93.44 ± 11.78	84.56 ± 9.55	−4.16	<0.001
Triglycerides (g/dL)	155.44 ± 90.31	146.61 ± 71.59	−0.871	0.39
HDL-cholesterol (g/dL)	52.39 ± 13.52	55.97 ± 10.99	1.533	0.134
**MetS indicators prevalence**				
Elevated waist circumference	26 (68.4)	16 (42.1)		0.002
Elevated fasting glucose	10 (27.0)	6 (16.2)		0.219
Elevated triglycerides	16 (44.4)	16 (44.4)		1
Reduced HDL-cholesterol	9 (25.0)	7 (19.4)		0.688
Elevated blood pressures	22 (57.9)	20 (52.6)		0.688
**MetS prevalence**				0.344
Less than 3 components	19 (52.8)	23 (63.9)		
3 or more components *	17 (47.2)	13 (36.1)		
**MetS score(z-score)**				
Male (*n* = 31)	−0.61 ± 3.35	−2.32 ± 2.55	−3.586	0.001
Female (*n* = 10)	−3.99 ± 2.05	−5.50 ± 2.19	−2.620	0.028
**Individual factors**				
General health status ^d^				0.109
Bad	30 (78.9)	26 (68.4)		
Good	8 (21.1)	12 (31.6)		
General self-efficacy	29.08 ± 2.76	30.05 ± 2.94	−1.81	0.079
Stress level (1–14)	9.08 ± 1.62	8.86 ± 1.67	0.796	0.431
General (1–7)	4.11 ± 0.99	3.86 ± 1.03	1.222	0.23
Work-related (1–7)	4.97 ± 0.90	5.00 ± 1.03	−0.19	0.85
Knowledge of MetS (0–6)	2.56 ± 1.73	2.71 ± 1.27	−0.482	0.632
Physical activity (per week)				0.376
No	14 (34.1)	9 (22.0)		
1–2 times	20 (48.8)	22 (53.7)		
≥3 times	7 (17.1)	10 (24.4)		
Sleep quantity (h) (3–10)				0.508
Others	29 (78.4)	26 (70.3)		
Good sleep (7 or 8 h)	8 (21.6)	11 (29.7)		
Diet (scores) (0–10)	4.47 ± 1.86	4.42 ± 2.38	0.151	0.881
**Organizational factors**				
Organizational commitment	6.22 ± 0.71	6.14 ± 1.03	0.572	0.571
Job satisfaction ^e^				0.581
Not satisfied	14 (37.8)	17 (45.9)		
Satisfied	23 (62.2)	20 (54.1)		
**Job stress**				
Job demand	57.43 ± 9.58	53.83 ± 10.50	2.053	0.047
Insufficient job control	56.98 ± 11.87	63.29 ± 13.68	−2.6	0.013
Inadequate social support	62.46 ± 11.82	67.57 ± 16.43	−1.667	0.104
Job insecurity	39.64 ± 21.28	42.34 ± 17.83	−0.758	0.454
Organizational system	55.86 ± 12.69	54.95 ± 12.02	0.384	0.703
Lack of rewards	55.86 ± 12.69	61.56 ± 14.49	−2.522	0.016
Occupational climate	34.91 ± 17.77	31.08 ± 12.98	1.432	0.161

SD = Standard deviation; BMI = Body mass index; HDL-cholesterol = high-density lipoprotein cholesterol; MetS = metabolic syndrome. * Metabolic syndrome was defined as having at least three of five components. ^a^ Numbers for characteristics do not total the same number due to missing data. ^b^ Paired *t*-tests for continual variables between groups; McNemar tests were conducted for categorical variables between groups. ^c^ *p* value for McNemar test or paired *t*-test. ^d^ Bad: very poor, poor, fair; Good: good, very good. ^e^ Not satisfied: Very dissatisfied, dissatisfied, fair; Satisfied: satisfied, very satisfied.

## References

[1] Mottillo S., Filion K.B., Genest J., Joseph L., Pilote L., Poirier P., Rinfret S., Schiffrin E.L., Eisenberg M.J. (2010). The metabolic syndrome and cardiovascular risk: A systematic review and meta-analysis. J. Am. Coll. Cardiol..

[2] Galassi A., Reynolds K., He J. (2006). Metabolic syndrome and risk of cardiovascular disease: A meta-analysis. Am. J. Med..

[3] Malik S., Wong N.D., Franklin S.S., Kamath T.V., Gilbert J., Pio J.R., Williams G.R. (2004). Impact of the metabolic syndrome on mortality from coronary heart disease, cardiovascular disease, and all causes in United States adults. Circulation.

[4] Gami A.S., Witt B.J., Howard D.E., Erwin P.J., Gami L.A., Somers V.K., Montori V.M. (2007). Metabolic syndrome and risk of incident cardiovascular events and death: A systematic review and meta-analysis of longitudinal studies. J. Am. Coll. Cardiol..

[5] Lim S., Shin H., Song J.H., Kwak S.H., Kang S.M., Yoon J.W., Choi S.H., Cho S.I., Park K.S., Lee H.K. (2011). Increasing prevalence of metabolic syndrome in Korea. Diabetes Care.

[6] Mozumdar A., Liguori G. (2011). Persistent increase of prevalence of metabolic syndrome among US adults: NHANES III to NHANES 1999–2006. Diabetes Care.

[7] Nestel P., Lyu R., Low L.P., Sheu W.H.-H., Nitiyanant W., Saito I., Tan C.E. (2007). Metabolic syndrome: Recent prevalence in East and Southeast Asian populations. Asia Pac. J. Clin. Nutr..

[8] Ministry of Health and Welfare Current State and Issues of Chronic Diseases: Chronic Disease Factbook 2015. http://www.cdc.go.kr/CDC/notice/CdcKrIntro0504.jsp?menuIds=HOME001-MNU1154-MNU0005-MNU2572-MNU0110&cid=65024.

[9] Organization for Economic Cooperation and Development Employment Rate 2016. https://data.oecd.org/emp/employment-rate.htm#indicator-chart.

[10] Organization for Economic Cooperation and Development Average Annual Hours Actually Worked per Worker 2016. https://data.oecd.org/emp/hours-worked.htm#indicator-chart.

[11] Chae D.H., Kim S.H., Lee C.Y. (2013). A study on gender differences in influencing factors of office workers’ physical activity. J. Korean Acad. Community Health Nurs..

[12] Lee J.-M., Kwon Y.-S., Paek K.-S. (2014). The relationship between lifestyle and health status among white collar workers in a community. J. Digit. Converg..

[13] Kim E., Oh S.W. (2012). Gender differences in the association of occupation with metabolic syndrome in Korean adults. Korean J. Obes..

[14] Lee W., Yeom H., Yoon J.H., Won J.U., Jung P.K., Lee J.H., Seok H., Roh J. (2016). Metabolic outcomes of workers according to the international standard classification of occupations in Korea. Am. J. Ind. Med..

[15] Dalle Grave R., Calugi S., Centis E., Marzocchi R., El Ghoch M., Marchesini G. (2010). Lifestyle modification in the management of the metabolic syndrome: Achievements and challenges. Diabetes Metab. Syndr. Obes..

[16] Lin K.M., Chiou J.Y., Ko S.H., Tan J.Y., Huang C.N., Liao W.C. (2015). Modifiable lifestyle behaviors are associated with metabolic syndrome in a Taiwanese population. J. Nurs. Scholarsh..

[17] Carnethon M., Whitsel L.P., Franklin B.A., Kris-Etherton P., Milani R., Pratt C.A., Wagner G.R. (2009). Worksite wellness programs for cardiovascular disease prevention. Circulation.

[18] Centers for Disease Control and Prevention Workplace Health Promotion. http://www.cdc.gov/workplacehealthpromotion/health-strategies/index.html.

[19] Beresford S.A., Locke E., Bishop S., West B., McGregor B.A., Bruemmer B., Duncan G.E., Thompson B. (2007). Worksite study promoting activity and changes in eating (PACE): Design and baseline results. Obesity.

[20] Harris J.R., Hannon P.A., Beresford S.A., Linnan L.A., McLellan D.L. (2014). Health promotion in smaller workplaces in the United States. Annu. Rev. Public Health.

[21] McLeroy K.R., Bibeau D., Steckler A., Glanz K. (1988). An ecological perspective on health promotion programs. Health Educ. Behav..

[22] Bronfenbrenner U. (1977). Toward an experimental ecology of human development. Am. Psychol..

[23] Ryu H., Kim Y., Lee J., Yoon S.-J., Cho J.-H., Wong E., Jung J. (2016). Office workers’ risk of metabolic syndrome-related indicators: A 10-year cohort study. West. J. Nurs. Res..

[24] Ryu H., Chin D.L. (2016). Factors associated with metabolic syndrome among Korean office workers. Archiv. Environ. Occup. Health.

[25] Wandersman A., Valois R., Ochs L., de la Cruz D.S., Adkins E., Goodman R.M. (1996). Toward a social ecology of community coalitions. Am. J. Health Promot..

[26] Grundy S.M., Cleeman J.I., Daniels S.R., Donato K.A., Eckel R.H., Franklin B.A., Gordon D.J., Krauss R.M., Savage P.J., Smith S.C. (2005). Diagnosis and management of the metabolic syndrome. Circulation.

[27] Lee S.Y., Park H.S., Kim D.J., Han J.H., Kim S.M., Cho G.J., Kim D.Y., Kwon H.S., Kim S.R., Lee C.B. (2007). Appropriate waist circumference cutoff points for central obesity in Korean adults. Diabetes Res. Clin. Pract..

[28] Schwarzer R., Jerusalem M., Weinman J., Wright K., Johnston M. (1995). Generalized Self-Efficacy scale. Measures in Health Psychology: A User’s Portfolio. Causal and Control Beliefs.

[29] Lambert S.J., Hopkins K. (1995). Occupational conditions and workers’ sense of community: Variations by gender and race. Am. J. Commun. Psychol..

[30] Chang S.J., Koh S.B., Kang D., Kim S.A., Kang M.G., Lee C.G., Chung J.J., Cho J.J., Son M., Chae C.H. (2005). Developing an occupational stress scale for Korean employees. Korean J. Occup. Environ. Med..

[31] Johnson J.L., Slentz C.A., Houmard J.A., Samsa G.P., Duscha B.D., Aiken L.B., McCartney J.S., Tanner C.J., Kraus W.E. (2007). Exercise training amount and intensity effects on metabolic syndrome (from studies of a targeted risk reduction intervention through defined exercise). Am. J. Cardiol..

[32] Takimoto M., Kibushi M., Okoshi Y., Nakagawa T., Irokawa M., Yakura H., Tanaka M., Matsuda S. (2008). Body weight reduction program for metabolic syndrome. Asian Pac. J. Dis. Manag..

[33] Maruyama C., Kimura M., Okumura H., Hayashi K., Arao T. (2010). Effect of a worksite-based intervention program on metabolic parameters in middle-aged male white-collar workers: A randomized controlled trial. Prev. Med..

[34] Matsuo T., So R., Shimojo N., Tanaka K. (2015). Effect of aerobic exercise training followed by a low-calorie diet on metabolic syndrome risk factors in men. Nutr. Metabol. Cardiovasc. Dis..

[35] Mache S., Jensen S., Linnig S., Jahn R., Steudtner M., Ochsmann E., Preuß G. (2015). Do overweight workers profit by workplace health promotion, more than their normal-weight peers? Evaluation of a worksite intervention. J. Occup. Med. Toxicol..

[36] Engbers L.H., van Poppel M.N., Paw M.J.C.A., van Mechelen W. (2005). Worksite health promotion programs with environmental changes: A systematic review. Am. J. Prev. Med..

[37] Lemon S.C., Zapka J., Li W., Estabrook B., Rosal M., Magner R., Andersen V., Borg A., Hale J. (2010). Step ahead: A worksite obesity prevention trial among hospital employees. Am. J. Prev. Med..

[38] Muse L., Harris S.G., Giles W.F., Feild H.S. (2008). Work-life benefits and positive organizational behavior: Is there a connection?. J. Organ. Behav..

